# Enabling Technology for Supramolecular Chemistry

**DOI:** 10.3389/fchem.2021.774987

**Published:** 2021-11-15

**Authors:** Katie Ollerton, Rebecca L. Greenaway, Anna G. Slater

**Affiliations:** ^1^ Department of Chemistry and Materials Innovation Factory, University of Liverpool, Liverpool, United Kingdom; ^2^ Department of Chemistry, Molecular Sciences Research Hub, Imperial College London, London, United Kingdom

**Keywords:** supramolecular chemistry, self-assembly, flow chemistry, high-throughput screening, automation, reaction monitoring

## Abstract

Supramolecular materials–materials that exploit non-covalent interactions–are increasing in structural complexity, selectivity, function, stability, and scalability, but their use in applications has been comparatively limited. In this Minireview, we summarize the opportunities presented by enabling technology–flow chemistry, high-throughput screening, and automation–to wield greater control over the processes in supramolecular chemistry and accelerate the discovery and use of self-assembled systems. Finally, we give an outlook for how these tools could transform the future of the field.

## Introduction

Supramolecular chemistry exploits weak, reversible interactions to form complex structures from simpler components (Lehn, 1988; Vantomme and Meijer, 2019). Two key tenets of supramolecular chemistry are host-guest molecular recognition and self-assembly (Davis et al., 2002), which have both become broad disciplines (Albrecht, 2007). Both concepts are ubiquitous in nature: the enzyme-substrate complex, base stacking of DNA and assemblies of virus cages are only a few examples (Whitesides et al., 1991). It is clear to see why chemists want to harness these principles in artificial structures for applications such as artificial enzymes, drug delivery systems, innovative materials, and more.

Huge progress has been made in the function, selectivity, and efficiency of artificial supramolecular systems. The diversity of materials and structures, including those that exploit mechanical bonds [catenanes (Gil-Ramírez et al., 2015), rotaxanes (Yang et al., 2019), knots (Fielden et al., 2017)], host-guest interactions [macrocycle (Liu et al., 2017), cage structures (Hasell and Cooper, 2016)], and framework or soft materials [metal-organic frameworks (MOFs) (Zhou and Kitagawa, 2014; Jiao et al., 2019), hydrogen-bonded organic frameworks (HOFs) (Yusov et al., 2021), supramolecular polymers (Yang et al., 2015) and gels (Weiss, 2014)], is ever-increasing, as is our ability to design and use these structures. As such, promising applications are emerging: porous supramolecular materials have potential in carbon capture (Huck et al., 2014), self-healing polymers could produce materials with enhanced recyclability (Song et al., 2019), and glucose binders could transform how diabetes is managed (Tromans et al., 2019), to name just a few.

However, the formation of supramolecular structures often presents challenges, which in turn limits their wide-spread use. Both non-covalent reversible interactions and reversible covalent reactions can be difficult to control due to their sensitivity to environmental conditions, and often a multitude of possible products are formed as a result (Wu and Isaacs, 2003). Solvent effects (Würthner, 2021) can be unpredictable and lead to unexpected outcomes (Little et al., 2014; Zhang et al., 2015). The use of high-dilution conditions, templating strategies, long reaction times or slow addition of reagents, and/or complex synthetic routes can help overcome these challenges (Marti-Centelles et al., 2015), but in turn limits the scalability of the process. Predictions of which assembly pathway will be followed, or the likely success of the process, can be aided using crystal engineering (Desiraju, 1997) or guided by computation (Greenaway and Jelfs, 2020), but the targeted design of materials with desirable properties can still be a long and restrictive process. Discoveries have generally been made *via* rational and iterative design techniques (Hong et al., 2015), often based on known structures, trial-and-error, or even serendipity (Winpenny, 2002; Saalfrank et al., 2008). However, these approaches limit the scope of possible accessible materials and leave vast areas of chemical space unexplored.

How, then, do supramolecular chemists approach these challenges? Standard tools for the chemist–i.e., round bottomed flasks–do not offer either fine control over environmental parameters (concentration, diffusion and mixing, temperature gradients), particularly on a large scale (Nomura et al., 1996; Kuśmierek and Świątkowski, 2015), or rapid exploration of chemical parameter space. There is therefore a need for tools that enable: 1) greater control over the formation of reversible and/or non-covalent interactions; 2) faster and more extensive exploration of chemical space; and 3) scalable, efficient, and “green” synthesis. This Minireview will explore two complementary approaches to solving these issues: flow chemistry and high-throughput automation, both of which fall under the umbrella of “enabling technology.”

Flow chemistry involves conducting a reaction in a continuous stream in a tube or microreactor (Myers et al., 2014; Plutschack et al., 2017). Flow processes are used extensively in industry; the majority of commodity chemicals are continuously manufactured (Glaser, 2015; Porta et al., 2016; Baumann et al., 2020). Flow chemistry offers benefits such as improved safety and control of reactions, access to a wider range of reaction conditions, easier scale-up, and potential savings in energy use and wastage (Newman and Jensen, 2013). Early adopters of continuous flow chemistry within the supramolecular community used microfluidic chips to influence process outcome (Whitesides, 2006; Zhang et al., 2012; Foster et al., 2015; Parker et al., 2015; Yu et al., 2015; Gong et al., 2016); significant progress in flow technology has since opened a wealth of new opportunities for the field (Arnon et al., 2016; Fang et al., 2018; Cohen-Gerassi et al., 2020; Méndez-Ardoy et al., 2020; Khoeini et al., 2021; Puigmarti-Luis et al., 2021).

High-throughput screening (HTS) enables reactions or processes to be rapidly carried out in parallel, potentially running thousands of samples simultaneously (McNally et al., 2011). Whilst HTS may have been previously affected by a bottleneck in slower dispensing, and analytical or data-processing steps, its growing success has been enabled by the development of automated experimentation platforms and technologies such as liquid and solid handling devices, robotics, autosamplers, and data-analyst software or scripts (Carson, 2020). It has been widely adopted within the pharmaceutical industry (Mennen et al., 2019) and is now finding its use in many other chemical fields.

**FIGURE 1:**
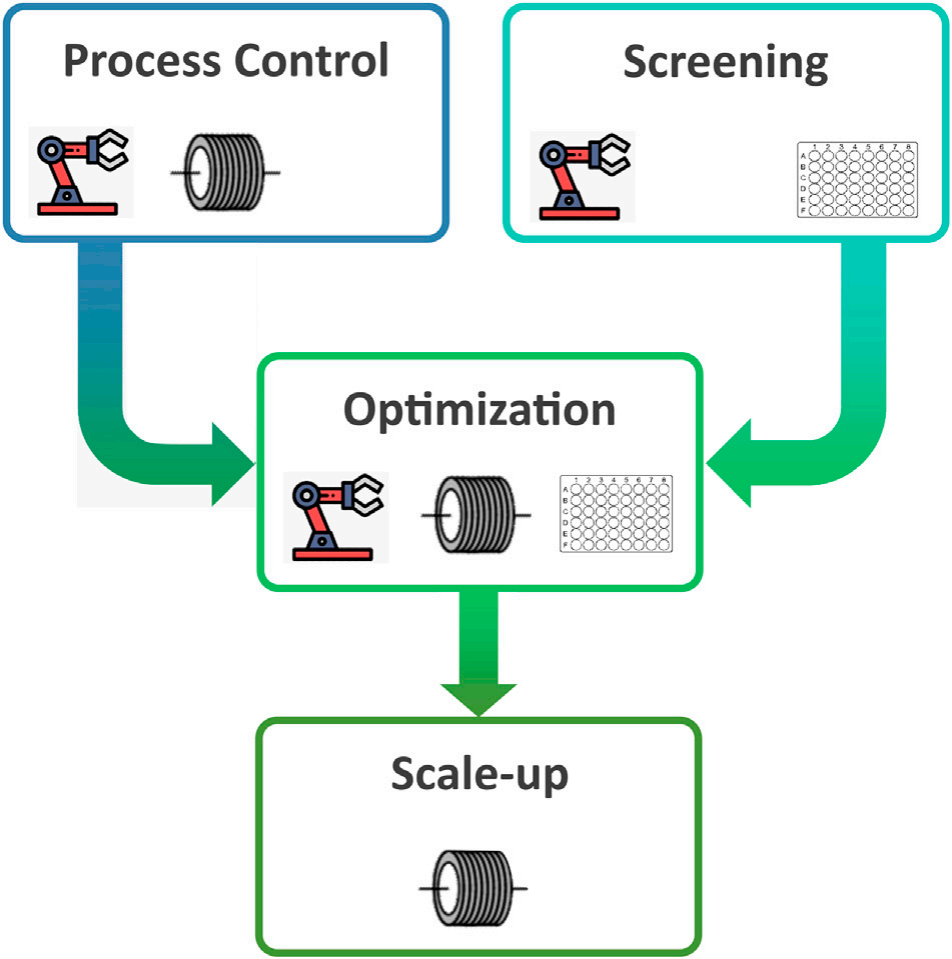
Typical processes involved in supramolecular system discovery and exploitation that can be enhanced using automation, flow chemistry, and/or high-throughput screening.

Although both technologies have had uptake in adjacent fields, there have been fewer reports on the use of flow or HTS in supramolecular chemistry. To showcase the benefits of such enabling technologies, this review will focus on the following four steps of development that are particularly relevant to supramolecular chemistry: enhanced process control, rapid screening and discovery of new structures, fast and/or automated optimisation, and facilitating scale-up (Figure 1).

## Enabling Technologies for the Stages of Development in Supramolecular Chemistry

### Enhanced Process Control

Supramolecular chemists seek to develop materials and systems made of multiple molecular building blocks where the whole is “more than the sum of its parts.” Many have exploited the reversibility of both non-covalent and dynamic covalent systems to form multicomponent structures under thermodynamic equilibrium (Whitesides et al., 1991; Rowan et al., 2002; Otto and Severin, 2007; Han et al., 2010; Okesola and Mata, 2018). Under fully reversible equilibrium conditions, the final outcome of such processes only depends on the molecular building blocks being used and the overall stability of the products: the thermodynamic product is often observed, representing the global energy minimum (Rowan et al., 2002). However, “out-of-equilibrium” structures have recently gained more attention (Ogi et al., 2014). For these systems, the reaction environment has a strong influence on the assembly pathway and the final structures obtained. Under non-equilibrium conditions, or where diffusion is slower than the rate of reaction, the impact of mixing efficiency or local concentration gradients can be substantial (Sevim et al., 2018). Controlling mixing is very difficult to achieve in a standard flask, but can be readily achieved under flow and microfluidic conditions (Nagy et al., 2012; Ward and Fan, 2015).

Microfluidic reactors–where reactions are carried out in channels <1 mm–offer unique control over the mixing of reagents in both space and time (“spatio-temporal control”) (Sevim et al., 2018). The degree of mixing can be much more finely controlled compared to batch, ranging from extremely turbulent flow and thus fast mixing to extremely slow: in the laminar flow regime, streams of fluids flow parallel to each other, creating a defined liquid-liquid interface between them (Brivio et al., 2006). Thus, specific assembly pathways can be deliberately targeted by controlling the flow regime, and obtaining non-equilibrium structures becomes easier in microfluidic environments, facilitating the targeted synthesis of materials with desired properties. Furthermore, in flow, a higher degree of control results in greater reproducibility and a consequent avoidance of off-target reactions due to poor mixing and local concentration gradients.

One recent example where this has been successfully exploited in supramolecular chemistry is the work of Numata et al. (2015b) who were able to synthesise porphyrin microfilms stabilized by an extended two-dimensional hydrogen bonding network. In contrast to batch conditions which formed an amorphous material, the use of microflow conditions enabled the formation of regular hydrogen-bonded networks leading to micron-sized multi-layered porphyrin sheets. This suggested that the microsheets could only be formed following the non-equilibrium kinetic pathway established under microflow conditions. Microfluidic conditions have also been used to access alternative self-assembled structures in a controlled manner in the field of organic conductors (Puigmartí-Luis et al., 2010), co-ordination polymers (Rubio-Martinez et al., 2016b), covalent organic frameworks (COFs) (Rodríguez-San-Miguel et al., 2016; Singh et al., 2018), and MOFs (Ameloot et al., 2011), and to control the hierarchical supramolecular assembly of perylene bisimides (Numata et al., 2015a), nanofibers (Numata and Kozawa, 2013), and amphiphiles (Numata et al., 2015c), with the latter enabling the formation of energetically unfavourable self-assembled structures under kinetic control.

Whilst control over reaction outcomes and the assembly process is a powerful tool, it is made more powerful if it can be rapidly applied to a wide range of systems. One potential drawback of continuous flow is the challenge of parallelising experiments, which currently limits throughput. HTS allows a much faster, and arguably more efficient, exploration of a wider chemical space, and can lead to the accelerated discovery of new structures. Efforts to parallelise or improve the throughput of flow experiments include microdroplet screening (Reizman et al., 2016), continuous variation of variables coupled within inline analysis (Aroh and Jensen, 2018), and multi-channel chip architectures (Headen et al., 2018), and it is likely that these approaches will become increasingly used to combine the benefits of enhanced control with rapid screening.

### Faster, Targeted Screening

Synthetic screening often results in a vast number of structures with only a select few being suitable for the application of interest. Fast and effective screening of the design space thus plays an important role in identifying materials and assemblies with desirable properties in supramolecular chemistry, and this is where HTS can offer its services.

A typical approach to HTS involves the use of multi-well microtiter plates and is often aided by the use of automated robotic platforms. For example, Greenaway et al. (2018) illustrated the advantages of employing an automated platform for HTS alongside computational analysis in the search for new organic cages. Overall, this led to the streamlined discovery of 33 new organic cages, with two forming bridged-catenane structures upon recrystallization, a new cage topology, highlighting the advantage of HTS in accelerating serendipitous discoveries. Their automated workflow has also been employed in the discovery of other supramolecular assemblies including an unsymmetrical organic cage (Berardo et al., 2018) and socially self-sorted organic pots (Greenaway et al., 2019). More recently, Cui et al. (2019) demonstrated how automated platforms can be used for polymorph screening, resulting in different HOFs being accessed. Additionally, Lin et al. (2021) demonstrated how HTS can screen for supramolecular diversification, resulting in a range of different hierarchical self-assemblies such as thin fibrils, helical ribbons, twisted ribbons, wide and thin ribbons, and macroscopic hydrogels.

Despite the potential advantages HTS offers, it has still not been widely adopted in the field of supramolecular chemistry. This could be due to factors such as the typically high initial cost of automated platforms, or challenges in finding suitable and equally high-throughput characterisation techniques for the supramolecular assemblies being targeted. However, with the availability of lower cost and open-source automated liquid handling platforms, and with several advances having been made in the HT characterisation of supramolecular materials (Greenaway et al., 2018; White et al., 2020), we expect these techniques to become more widely adopted in the field for screening and discovery of new “hits.”

### Rapid Optimization

Once a “hit” has been found, the next step in the process is to optimize for parameters such as yield, selectivity, performance, throughput, or stability; this process can take as long as the discovery phase. Here, enabling technologies offer the opportunity to shorten this timeline and assist the chemist in making key decisions to ensure the best chance of finding an optimal supramolecular system.

Flow chemistry enables real-time analysis, giving mechanistic insight that can be used to inform optimization. Intermediates can be isolated from the reaction stream and separately analysed to provide a better understanding of the reaction pathway and general self-assembly processes (Sagmeister et al., 2021). For example, recently Jones et al. (2021) exploited this to optimise the synthesis of a macrocyclic molecular hinge using a semi-continuous method informed by at-line analysis. Here, the fast heat transport possible in flow enabled a rapid temperature change mid-synthesis, leading to a threefold increase in yield and simultaneous reduction in reaction times.

HTS can also be used to screen many different parameters in parallel (e.g., reagent stoichiometries, solvents, catalysts, additives, and temperatures), and has been widely used in the pharmaceutical industry to optimise yield and selectivity (Shevlin, 2017; Mennen et al., 2019). This optimisation process can also be aided by incorporating Design of Experiments (DoE) to narrow down the number of reactions required. In addition, microfluidic platforms can also be employed for HTS which, when compared to automated platforms and microplates, can significantly reduce the quantities of reagents required for both screening and optimisations (Zhou et al., 2020). Whilst there are very few examples of applying flow, microfluidics, or automated platforms for high-throughput screening in supramolecular chemistry, there is no reason why these approaches could not be adopted for the field.

It is worth noting that whilst HTS can enable a large amount of the design space to be investigated, it is not desirable to fully empirically explore all precursor combinations or reaction conditions to ensure the optimal conditions are selected. By combining HTS and flow chemistry with machine learning algorithms and Bayesian optimisations, and *in situ* analysis techniques, this process can be streamlined by using closed-loop autonomous screening to optimise towards a target parameter or property (Zhou et al., 2017; Bédard et al., 2018; Clayton et al., 2019; Clayton et al., 2020). A recent example that highlights this in the area of supramolecular chemistry is the work of Cronin and co-workers who used a closed-loop autonomous “chemical robot” to explore a large combinatorial space for the discovery of coordination architectures (Porwol et al., 2020). The group calculated that it would take 4 × 10^4^ experiments to fully explore the variables chosen, and therefore proposed an autonomous decision-making approach to explore this space as rapidly as possible, discovering four new coordination complexes in the process. This example highlights the importance of decision-making in screening large parameter spaces: the task of deciding “what to explore,” or “what to explore next,” normally taken by the chemist, is a critical point that determines the success or failure of the experiment as well as how fast that point is reached. Emerging research in algorithms for process space exploration, growing from the established science of DoE approaches, are yielding incredible advances in discovery speed in adjacent fields and are likely to make a similar impact in supramolecular chemistry, especially if suitable conditions for scale-up can also be identified.

### Scale-Up

Despite the activity in the field, currently, there are only a few supramolecular materials that are produced for commercial purposes. The many issues surrounding the scale-up process (e.g., large amounts of organic solvents, long reaction times, poor reproducibility) have hindered this transition, preventing structures from being viably used for the many promising applications they present (Rubio-Martinez et al., 2017).

Continuous flow reactors are one of the latest technologies being employed to produce supramolecular structures at scale. Their large surface area-to-volume ratio allows more efficient mass and heat transfer, giving safer and faster reactions. Improvements on the reproducibility and product quality can be obtained due to the higher level of control over the reaction. Flow reactors also generally require less solvent compared to traditional batch reactions, driving down the cost and waste which are all factors that need to be considered for an industrial scale process (Rubio-Martinez et al., 2017).

Recently, flow chemistry has been used to optimize the yield, selectivity, and limited scale-up, of both macrocyclic (Bogdan and James, 2010; Bogdan and James, 2011; Bedard et al., 2013; Bédard et al., 2015; Lucke et al., 2016; Morin et al., 2019; Jones et al., 2021; Seemann et al., 2021) and cage (Briggs et al., 2015; Kitchin et al., 2015) supramolecular structures. For example, Bédard et al. synthesized medicinally relevant (Marsault and Peterson, 2011) macrocyclic lipids under continuous flow, with yields of up to 97% in short reaction times (Bedard et al., 2013). They were also able to increase the reaction to a multigram scale for some macrocycles; high yields were retained without the need for re-optimisation. Similarly, Briggs et al. (2015) translated the synthesis of porous organic cages (POCs) from batch to flow, resulting in greatly reduced reaction times and reduced solvent use, offering a continuous method of scale-up. It should be acknowledged, however, that there is still significant method development required to achieve the multi-kilo or tonne scale needed for many industrial applications, and here collaboration with process chemists and engineers is essential.

Flow has also assisted in the scale-up of MOFs (Munn et al., 2015), which have promising applications in gas storage, gas separation and heterogeneous catalysis, but are typically produced by solvothermal batch synthesis that is challenging on a large scale (Czaja et al., 2009). For example, Rubio-Martinez et al. (2016a) were able to scale-up the synthesis of the aluminium fumate (Al-Fum) MOF from a laboratory to a pilot-plant system by more than 2 orders of magnitude giving an increased space-time-yield (STY) of 97,159 kg m^−3^ day^−1^. Since then, significant progress has been made towards the commercial synthesis of MOFs; for example, a continuous hydrothermal synthesis is now being used by Promethean Particles, who commercially produce nine different MOFs and porous structures (Munn et al., 2015).

It is also worth noting that scale-up of supramolecular materials has also been demonstrated under mechanochemical continuous flow, namely using twin-screw extrusion (TSE), which can reduce the solvent requirement for synthesis even further. For example, both MOFs and POCs have been formed on scale with very little to no solvent using TSE (Crawford et al., 2015; Egleston et al., 2020; Casaban et al., 2021). Finally, there have also been promising developments in combining flow reactors and microwave heating for the process intensification of MOF formation (Laybourn et al., 2019). It is clear that the materials chemist has a number of tools at their disposal to ensure compounds can be made at-scale, and we anticipate this will become more widespread in supramolecular chemistry.

## Conclusion and Outlook

**FIGURE 2:**
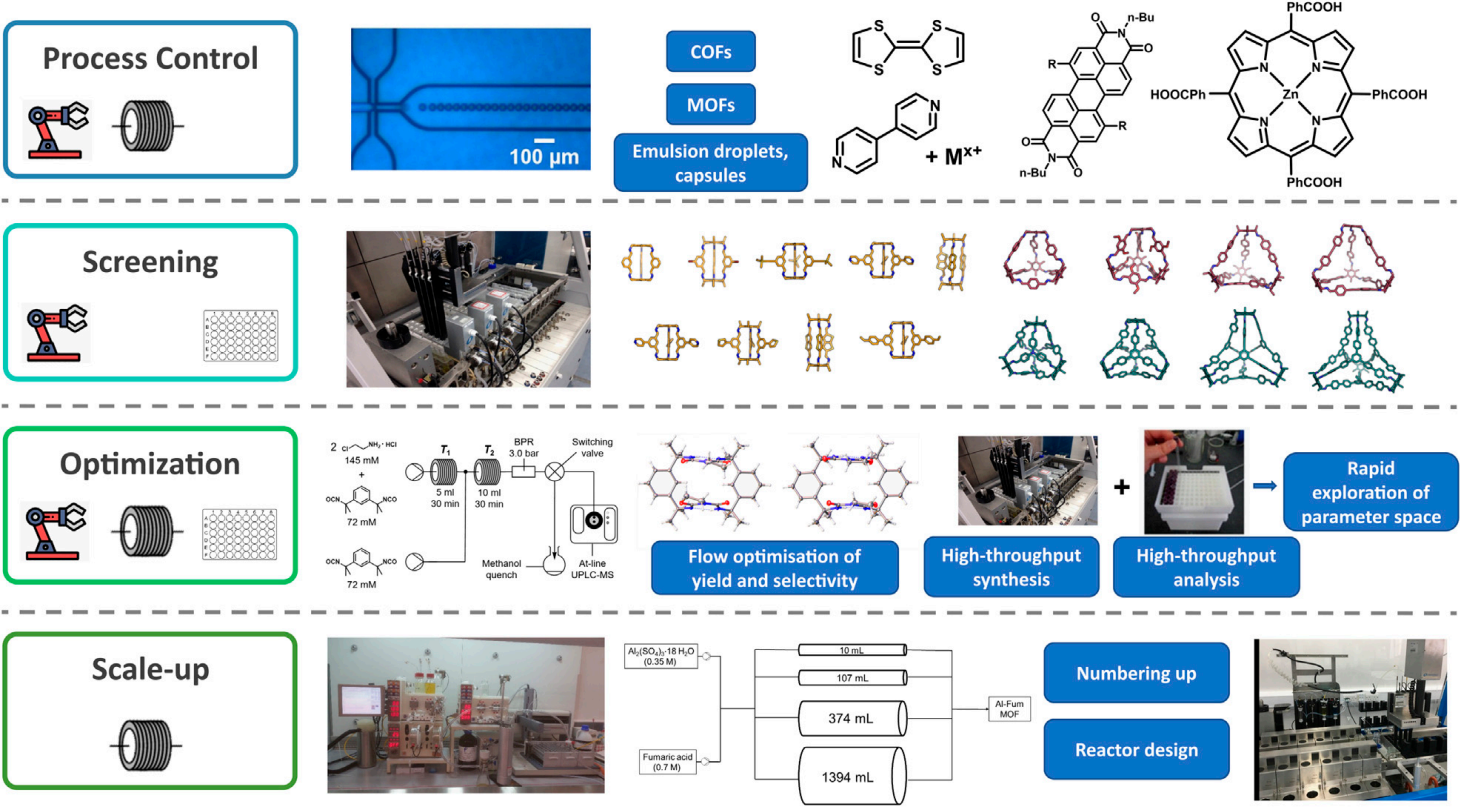
Examples of supramolecular species and precursors and self-assembly processes that have been examined or enhanced using enabling technology for process control (Puigmartí-Luis et al., 2010; Numata et al., 2015a; Rubio-Martinez et al., 2016b; Gong et al., 2016; Thorne et al., 2019; Puigmarti-Luis et al., 2021, This figure contains an image of Figure 2a from Thorne et al. that has been used to make this composite figure under the terms of a Creative Commons Attribution 4.0 International License https://creativecommons.org/licenses/by/4.0/), screening (Greenaway et al., 2018), optimisation (Jones et al., 2021, The flow diagram and crystal structures in the optimization panel have been reused from Figure 2 and 11 from https://pubs.acs.org/doi/abs/10.1021/jacs.1c02891 respectively with permission from the ACS. Further permission related to the material excerpted should be redirected to the ACS), and scale up (Briggs et al., 2015; Rubio-Martinez et al., 2016a).

Emerging examples of the use of enabling technology in supramolecular chemistry showcase the potential impact on the field: from faster discovery and screening to efficient optimisation, analysis, and scale-up (Figure 2). Commercial flow reactors, robots and high-throughput work stations are increasingly available alongside low-cost, home-build options (Baas and Saggiomo, 2021), meaning these enabling technologies are becoming more accessible to research laboratories. It is likely that as this availability of technology increases, it will become more integrated in research laboratory procedures and industrial manufacturing. If these technologies are to become mainstream, then training the chemists of the future will become vital. Examples of pre-designed flow chemistry experiments for potential use in undergraduate teaching laboratories (König et al., 2013; Leibfarth et al., 2018; Kuijpers et al., 2021) represent the acknowledgement of a changing skill set required of graduate chemists.

It should also be noted, that whilst this review has focussed on the benefits of enabling technology, it is now frequently coupled with computation to carry out prior predictions and subsequent analysis. Growing examples can already be seen of computational predictions in areas such as organic cages (Berardo et al., 2020), photocatalysts (Singh et al., 2015), and drug molecules (Yu et al., 2020). The likely outlook for the future of chemistry, including supramolecular chemistry, is a hybrid approach of automated experimentation coupled with computation, whether that is using computation to first narrow down the design space, or using HTS to collect large amounts of robust data to feed into data-led computational approaches and machine learning algorithms. It seems certain that the benefits these tools offer will play an increasing role in supramolecular chemistry, from screening to scale-up.
